# INTERNET USE AND SOCIAL ISOLATION: THE SIGNIFICANCE OF LIFE TRANSITIONS

**DOI:** 10.1093/geroni/igz038.698

**Published:** 2019-11-08

**Authors:** Hyunju Shim, Jennifer A Ailshire, Eileen M Crimmins

**Affiliations:** 1 University of Southern California, Los Angeles, United States; 2 University of Southern California, Los Angeles, California, United States

## Abstract

Social isolation has emerged as a significant public health risk for older people, and can be triggered by a number of life transitions in old age, such as retirement, bereavement, and divorce. Using the Health and Retirement Study (HRS), the current study aims to examine how internet use is associated with significant life transitions in marital and employment status in a longitudinal analysis. Results showed that using the internet was associated with lower social isolation and depression for people who became separated or divorced, but no significant interaction was observed for people who became widowed. On the other hand, becoming unemployed and using the internet was associated with more depressive symptoms. The results indicate that the link between psychological well-being and use of internet differs depending on the context of life transitions and may not have universal benefits for reducing social isolation.

